# The Surgical Treatment of Gastric Ulcer

**Published:** 1904-06-04

**Authors:** 


### THE SURGICAL TREATMENT OF GASTRIC ULCER.

Signs are not wanting of a growing inclination on the part of the medical profession generally to accept, possibly with some reservations, the dicta of the comparatively small number of surgeons who have of recent years been so strongly advocating an earlier resort to surgery in certain gastric diseases, notably in non-malignant gastric ulcer. Increased experience has shown that many of the objections raised against operative measures in the various conditions due to gastric ulcer have not proved valid in practice. A common source of fallacy has been the drawing of inferences from post-mortem appearances without due allowance being made for the difference between living and dead structures. Thus it has been urged that if gastrotomy be done in cases of hæmorrhage from the stomach, it may be impossible to detect the spot from which the blood is escaping; and the main foundation for this statement appears to be that in a certain percentage of cases of death from gastric hæmorrhage it is impossible, or very nearly so, to find any lesion post-mortem. As Mr. Mansell Moullin[1] points out, the inner surface of the stomach presents a very different appearance during life to that which is seen in the post-mortem room. In the living, the mucous membrane is soft and thick, very vascular, and bright red in colour. If there is any abrasion or ulcer, the edges around it are swollen and œdematous, and, moreover, blood may readily be observed issuing from the inflamed spot. After death the swelling disappears, the mucous membrane becomes uniform and flat, and the abraded surface may become so nearly level with the rest, that the difference can be scarcely detected. Mr. Moullin states that in upwards of fifty cases on which he has operated, when the bleeding was recent, he has not failed to find the source of hæmorrhage. In the light of this experience he maintains that a surgeon should be consulted in every case of hæmatemesis, whether arising, from gastric ulcer or not, as soon as it has become evident that the loss of blood is really serious, and is not checked by medical treatment. If the patient has not been allowed to bleed nearly to death previously he ought not to die from the operation. By opening the stomach on its anterior wall, and holding apart the edges of the opening by means of four Spencer Wells' forceps, all the interior that lies near can be easily inspected; that which lies a little further off can be seen by bringing with the fingers portion after portion of the wall before the opening, and by introducing a full-sized vaginal speculum into the stomach, the whole of the rest of the mucous surface can be minutely inspected, and the bleeding point when found can be readily secured. Satisfactory as such a procedure is, it cannot of course prevent the subsequent development of other ulcers in the stomach, and recurrence does sometimes take place.

In hæmorrhage from chronic ulcer the reasons for operating are even more cogent than in the case of acute lesions; for, as museum and post-mortem specimens show, the bleeding in a large proportion of the former cases comes from an artery, and is therefore unlikely to cease in response to purely medical measures. In addition, an operation affords the chance of curing the patient of his ulcer which otherwise might persist for years, causing vomiting and pain the whole time, and undermining the patient's health, while entailing on him the constant risks of hæmorrhage, perforation, perigastric abscess, and peritonitis. Another point to be remembered is that the longer a chronic ulcer remains the greater is the difficulty in successfully treating it either by surgical or medical measures. The ideal treatment for chronic ulcer is excision, but when this is impossible, as it is when the pylorus or lesser curvature is involved, all that can be done is to try and relieve the symptoms by performing gastro-jejunostomy.

[1] Clinical Journal, May 25.

